# Design and Development of ‘Diet DQ Tracker’: A Smartphone Application for Augmenting Dietary Assessment

**DOI:** 10.3390/nu15132901

**Published:** 2023-06-27

**Authors:** Subeg Singh Mahal, Christopher Kucha, Ebenezer M. Kwofie, Michael Ngadi

**Affiliations:** 1 Department of Bioresource Engineering, McGill University, Macdonald Campus, 21111 Lakeshore Road, Ste-Anne-de-Bellevue, QC H9X 3V9, Canada; subeg.mahal@mail.mcgill.ca (S.S.M.); christopher.kucha@uga.edu (C.K.); ebenezer.kwofie@mcgill.ca (E.M.K.); 2 Department of Food Science and Technology, University of Georgia, 100 Cedar St., Athens, GA 30602, USA

**Keywords:** diet diversity, dietary assessment, mobile phone app

## Abstract

The purpose of the current study was to describe the design, development, and validation of the ‘Diet DQ Tracker’. The ‘Diet DQ Tracker’ is the first self-administered smartphone app designed to collect dietary data for diet diversity indicators. The main objective of the app was to replace the traditional methods of dietary data collection, such as in-person or telephone 24 h recall via pen and paper questionnaire or tablets. The real-time meal recording, extensive food database, and automatic score calculations and visualizations for MDD-W, IYCF-MDD, and HDDS have the potential to overcome the drawbacks of 24 h recalls. Recall depends on respondent memory, food expertise, and time consumption and demands skilled interviewers. Further, SAIN, LIM recommendations in the app prompt users to diversify diets with healthy foods. The pilot study determined the acceptability, feasibility, and relative validity of the ‘Diet DQ Tracker’ with a 24 h dietary recall. The results demonstrated minimal differences in dietary scores by both methodologies. The app, being convenient, easy to use, less time-consuming, and enjoyable, was preferred by the entire study sample over 24 h recall. The app will be continually updated with foods from different cultures for validating in large-scale studies. The future studies will help to improve the subsequent versions of the app.

## 1. Introduction

Global food security and nutrition continue to be at the forefront of the development agenda. Individual nutrition enhancement is being given much attention in meeting this global need. At the center of nutrition improvement, the United Nations agencies have been dedicating efforts towards improving the nutritional level of populations, especially in developing countries [1]. However, methods to capture the contribution of food systems adequately and systematically in nutrition research, although laudable, are currently not as efficient as they might be since many of them involve data collection with the pen and paper approach. These methods are time-consuming, sometimes subject to error, and can be cumbersome in terms of data entry and analysis [2]. Thus, the development of user-friendly methods such as smartphone technologies has a tremendous potential to capture detailed dietary information of individuals and groups much more efficiently.

The impact of agricultural interventions and programs to improve nutrition is based on the indicators. The choice of impact-assessing indicators is a crucial issue [3]. Studies in the last decade considered anthropometry as the most important indicator [4]. However, it has been established in recent years that dietary indicators are being used more often to assess the effectiveness of interventions. Dietary indicators are more sensitive to food availability and nutrition assessment [5]. The assessment of dietary intake, however, is a challenging endeavor. Quantitative dietary evaluation being complex, demands specialized skills. Numerous simple dietary indicators have been developed to address this issue, the most popular being diet-diversity indicators [4]. These diet-diversity indicators include Minimum Dietary Diversity for Women (MDD-W) and Minimum Dietary Diversity of Infant and Young Child Feeding Practices (IYCF-MDD) at the individual level and Household Dietary Diversity Score (HDDS) at the household level.

Nutritionists have long recognized that high-quality diets are influenced by dietary diversity. Most dietary guidelines around the world encourage increasing food diversity across and within food groups. Diet diversity promotes good health by providing an appropriate intake of vital nutrients [6]. The lack of food diversity is particularly problematic among poor populations in developing nations. Their carbohydrate rich diets are often lacking in animal products and fresh fruits and vegetables [7]. An adequate diet meets the individual’s energy and essential nutrient requirements. Diet-related chronic diseases and obesity have shifted the definition of quality diet towards moderation, proportionality, and diversity. Diet diversity is one of the key features of diet quality [6].

In public health research, identifying the usual diet is critical to improve the understanding of food intakes, diet quality, dietary determinants, and diet–disease interactions. The difficulties of establishing reliable dietary assessments have been widely researched and debated [8]. As a result, dietary assessment tools must be selected by considering the relative validity of different methodologies, respondent–researcher burden, and resources required for implementation [9]. In nutritional research, traditional methods such as 24 h (24 h) recalls, food records, food frequency questionnaires (FFQs), and interviews are the frequently practiced methodologies for determining food consumption intake [10]. However, these methods are hampered by limitations which affect the data collection and weaken the conclusions, especially if the error in reporting is not addressed to the maximum possible extent [11]. Normally, an interviewer determines an individual’s food intake in a specific recall period by asking questions from a questionnaire and recording the information on paper. Following this approach, the dietary diversity intake is calculated manually by using food composition tables or by using different software. This method entails numerous repeated steps that require a lot of human effort [12].

Further, in traditional retrospective methods such as 24 h recall, the interviewer probes the respondent to recall all food items consumed in the last 24 h, beginning at a specific reference point of time. Respondents often struggle differentiating between what they eat consistently and what they consumed the day before, resulting in inaccurate reporting [13]. Moreover, the inherent human capacity for event recall declines with time, initiating an hour after the diet intake [14]. The recall bias is directly proportional with the length of the recall period [15]. The accuracy of the recall can be strengthened if implemented multiple times within a 24 h window, consequently minimizing the error in reporting by shifting to a record-like approach from the recall approach [16]. The adoption of smartphone apps as dietary data collection tools presents a viable solution. Smartphones, being portable, enable respondents to collect self-administered dietary data right after consumption of the meal, thus overcoming the challenges encountered with recall bias. Another prevalent bias is the social-desirability bias. It is a pattern where participants tend to respond in a manner they believe would be deemed acceptable by others [17]. The greater the socialization in a survey, the greater the social-desirability bias [18]. In traditional dietary intake interviews, the social-desirability bias may result in over-reporting of healthy diets. In instances where respondents feel uncertain regarding the interviewer’s anticipated reaction, or when the recording process does not involve any interpersonal interactions, the responses tend to reflect the respondent’s genuine consumption habits [19]. The main contributors to social-desirability bias, such as the presence of an acquaintance or conversations with the interviewer [20], can be circumvented by transitioning from conventional practices to technology-driven methods. A smartphone app, ensuring respondent privacy and eliminating the need for interpersonal interactions, can aid in minimizing the social-desirability bias. Lastly, there is an escalating demand to augment precision while minimizing the cost and time related to data collection associated with traditional methodologies [21]. The conventional methodologies involve significant investment of resources for training, data collection, and presentation of results. To alleviate the effort, cost, and time involved in gathering and processing dietary intake data, dietary assessment studies are progressively leveraging technology [2].

Considering inherent difficulties such as dependency on memory, time consumption, food portion measurement, educated or experienced personnel, coding burden, food expertise, and other time-consuming chores [9], it has been suggested that utilizing mobile technologies may result in an improved assessment of food consumption and personalized nutritional feedback [22]. Further, recall bias and social-desirability bias can be addressed by recording dietary intake in real time via personal respondent accounts on smartphone apps. The use of mobile phones to assess and enhance the assessment of food consumption intake has been explored in several studies [23,24,25,26,27,28,29,30,31]. A review of 74 studies that used innovative technologies to improve diets found that, while traditional methods and new technologies often overlap in methodology, new technologies may deliver more accurate nutritional assessment by allowing cost- and time-efficient data collection and increased respondent acceptability [32].

Many mobile apps have been developed for distinct goals and validated against 24 h dietary recalls, such as ‘Eat and Track’ (EaT) [33], ‘My Meal Mate’ (MMM) [27], ‘electronic Dietary Intake Assessment’ (e-DIA) [34], ‘Easy Diet Diary’ [23], and ‘electronic Carnet Alimentaire’ (e-CA) [26]. However, as of the date of publication, none of the mobile apps available collects self-reported dietary data for diet diversity indicators at the population level in resource-poor settings.

The ‘Diet DQ Tracker’ is a self-administered smartphone app designed for iOS and Android to facilitate data collection in research studies for diet diversity indicators. The current study demonstrates design, development, and relative validity of the ‘Diet DQ Tracker’ with a 24 h interviewer-administered dietary recall. The ‘Diet DQ Tracker’ includes a customized database of 8777 food items from the USDA, Ethiopia, and Laos, which are grouped into relevant food groups using the diet-diversity indicators methodology. In addition, a desktop-run content management software application ‘NutriMetrics_CMS’ has been developed to facilitate database management and analysis of the results.

The study elaborates on the MDD-W, IYCF-MDD, and HDDS diet diversity indicators. The SAIN, LIM nutrient profiling system encourages users to include healthy foods in diverse diets. Following this, the food database, CMS, and third-party cloud services were linked together by developing an app architecture and Python algorithm. The pilot study evaluated the insignificant difference between diet diversity scores achieved by the ‘Diet DQ Tracker’ and an interviewer-administered 24 h recall. The user feedback survey determined the acceptability of the app as an effective dietary data collecting tool.

## 2. Materials and Methods

### 2.1. Diet Diversity Indicators

Deficits and differences in nutrition consistency at the individual and household levels have been known for a long time. Around 2 billion people worldwide suffer from micronutrient deficiency, a large portion of which is attributed to monotonous diets. Monotonous diets comprise nutrient-deficit staple crops [35,36]. As a result, the number of programmatic interventions aiming to improve diet diversity and nutrition has grown over time. Diet diversity indicators help to track the impact and progress of these interventions [37]. Consequently, the demand and need for diet diversity indicators has increased with time.

Further, the efficiency and efficacy of diet diversity indicators is directly linked with the methodology to collect dietary data. The frequently used method to measure diet diversity for individuals or households consists of evaluating the variety or number of different food groups (rather than food items) consumed in a specific period. The food groups used to calculate these indicators scores are mutually exclusive. In a 24 h period, each food group is counted once and weighed equally [38]. The higher the score, the higher the diversity in diet [6]. Depending on the level of measurement and intended purpose, the number of food groups may vary in different indicators [39]. At the individual level, these indicators reflect micronutrient adequacy whereas for households, diet diversity is an indicator of access to food, such as the household’s capacity to acquire food. In both theoretical and practical contexts, diet diversity indicators have been developed and used in many ways. Nevertheless, only a few simple indicators have been nurtured for population level use in resource-poor settings. These globally accepted indicators include Minimum Dietary Diversity for Women (MDD-W), Minimum Dietary Diversity of Infant and Young Child Feeding Practices (IYCF-MDD), and Household Dietary Diversity Score (HDDS) [4]. The successful implementation of these widely recognized indicators will aid in monitoring the effects and development of interventions aimed at enhancing dietary diversity, thereby improving general nutrition at the population level in resource-poor settings.

#### 2.1.1. Minimum Dietary Diversity for Women (MDD-W)

Physiological demands of pregnancy and lactation often make women of reproductive age (WRA) vulnerable to nutrition [7]. Sustainable development goals can be achieved by good nutrition in WRA. Historically, women, being responsible for food selection and preparation, have been the primary caregivers to children and elderly members of the household [40]. Diets including variety of food groups are critical for resisting malnutrition and foster better health in women and their offspring [39]. MDD-W is a dichotomous indicator of whether women 15–49 years of age have consumed at least five out of ten defined food groups the previous day or night [40]. The percentage of women between the ages of 15 and 49 who meet this minimum can be used as a proxy indicator for better micronutrient adequacy [40]. The following ten food groups count for the score: 1. Grains, white roots and tubers, and plantains; 2. Pulses (beans, peas, and lentils); 3. Nuts and seeds; 4. Dairy; 5. Meat, poultry, and fish; 6. Eggs; 7. Dark green leafy vegetables; 8. Other vitamin-A-rich fruits and vegetables; 9. Other vegetables; 10. Other fruits. The MDD-W score is a numeric value between 0 and 10, based on the consumption of food items or ingredients weighing equal to or more than 15 g from each of these food groups [40]. Along with these ten food groups, MDD-W includes six optional and two required categories that are not factored into the score calculation. The optional categories are: 1. Insects and other small protein foods; 2. Red palm oil; 3. Other oils and fats; 4. Savory and fried snacks; 5. Sweets; 6. Sugar-sweetened beverages. The required categories include: 1. Condiments and seasonings; 2. Other beverages and foods [40]. The recommended MDD-W threshold score for WRA in a 24 h period is 5. The WRA who consume at least five MDD-W food groups in a 24 h period are more likely to achieve their physiological needs and nutrition requirements with diet diversity.

#### 2.1.2. Infant and Young Child Feeding Practices—Minimum Dietary Diversity (IYCF-MDD)

The first thousand days of life are crucial for infant development [41]. Nutritional status and survival rate of children below two years of age are directly related to infant and young child feeding (IYCF) practices. Therefore, it is vital to improve IYCF practices in children aged 0–23 months, subsequently promoting nutrition, health, and development. Appropriate feeding practices should be implemented in a timely manner. In addition to breast feeding, infants ought to receive complementary foods, beginning at the age of 6 months. Minimum dietary diversity (MDD) is one of the eight core indicators of IYCF practices [42]. MDD is defined as whether children between the age group of 6 and 23 months have consumed food from five or more food groups out of the eight food groups in the last 24 h. The eight food groups that count for calculating IYCF-MDD score are: 1. Breast milk; 2. Grains, roots, and tubers; 3. Legumes and nuts; 4. Dairy products (milk, yogurt, cheese); 5. Flesh foods (meat, fish, poultry, and liver/organ meats); 6. Eggs; 7. Vitamin-A rich fruits and vegetables; 8. Other fruits and vegetables. The IYCF-MDD score is a numeric value between 0 and 8. Unlike MDD-W (15 g), for the IYCF-MDD score, there is no minimal quantity of food group required to be consumed [42]. The recommended IYCF-MDD threshold score for children aged 0–23 months in a 24 h period is 5. Therefore, the IYCF practices for better growth, nutrition, and development of children can be achieved by feeding them with 5 distinct MDD food groups in a 24 h period.

#### 2.1.3. Household Dietary Diversity Score (HDDS)

Dietary diversity is directly associated with food security and socio-economic status of households [43]. HDDS measures the economic capacity of households to acquire a variety of foods. HDDS calculates the number of different food groups (out of 12 food groups) consumed over a 24 h period. It is an attractive proxy indicator for a household’s food diversity. HDDS is focused on the desired outcome of improved food access, i.e., improved household food consumption [44]. The twelve food groups that are used for calculating HDDS are: 1. Cereals; 2. Roots and tubers; 3. Vegetables; 4. Fruits; 5. Meat, poultry, offal; 6. Eggs; 7. Fish and seafood; 8. Pulses, legumes, and nuts; 9. Milk and milk products; 10. Oil/fats; 11. Sugar/honey; 12. Miscellaneous. The HDDS score is a numeric value between 0 and 12 based on the consumption of food prepared at home consumed by any member of the household, irrespective of food quantity, from 12 food groups within a 24 h period. The HDDS measure provides an overview of a household’s food access across a 24 h period [44]. In contrast to MDD-W and IYCF-MDD, HDDS does not have a fixed threshold score for indicating adequate diet diversity of the household. However, an increase in the consumption of different HDDS food groups over a 24 h period by households would indicate their improved economic capacity to access food in resource-poor settings.

### 2.2. Diet Quality Indicator

Diet quality refers to a dietary pattern or indicator of diversity across key food groups as recommended by dietary guidelines [45]. It is determined by different factors such as the individual’s cultural and food environment, socio-economic status, household food preferences, and nutrition recommendations [46]. The term diet quality has emerged in the scientific literature over the last two decades, most frequently in nutritional epidemiology for assessing the efficacy of dietary interventions and dietary habits at the population level [47,48]. Diet quality has been introduced as a risk assessment tool for cancer, mortality, and cardiovascular disease risk prediction by researchers [49]. Until now, most of the dietary guidelines for health promotion have been largely based on data from single foods and nutrients. However, foods are not consumed in isolation. There has been an increased amount of attention to assess the quality of whole diet, including its complexity and how its different parts might interact with each other [50]. Diet quality measurement methods have evolved in recent years, with the emergence of a variety of indicators and scoring systems. In more refined diet quality scoring methods, protective dietary patterns and unfavorable food intakes are identified [45]. Overall, the diet quality measures both the quality and variety of an individual’s diet, allowing to assess the relationship between whole diet and state of health [45].

#### 2.2.1. SAIN, LIM

SAIN, LIM is a nutrient profiling system that categorizes food items into four classes. SAIN and LIM are two independent scores. SAIN is the score of nutritional adequacy of individual foods (positive or qualifying nutrients), and LIM is the score of nutrients to be limited (negative or disqualifying nutrients) [51,52].

The SAIN score’s limited number of nutrients (five primary nutrients and one optional nutrient) are determined by striking a balance between nutrients of public health importance, nutrient markers of essential food categories, and the requirement for a feasible nutrient profile in the research settings [53]. Protein, fiber, ascorbic acid, calcium, and iron are the primary nutrients, while vitamin D is an optional nutrient used to calculate the SAIN score. The SAIN score is the unweighted arithmetic mean of the percentage adequacy of five positive nutrients and one optional nutrient (Vit. D), calculated for 100 kcal of food. The nutrient_ip_ is the quantity (g, mg, or μg) of positive nutrient p in 100 g of food i, RV_p_ is the daily recommended value for nutrient p, and E_i_ is the energy content of 100 g of food i (in Kcal/100 g) [54].

(1) $$\begin{array}{c} {\mathrm{SAIN}}_{i}=\frac{\sum_{p=1}^{p=5}{ratio}_{ip}}{5}\times 100 \end{array}$$

with

(2) $$\begin{array}{c} {ratio}_{ip}=\left[\frac{{nutrient}_{ip}}{{RV}_{p}}\right]\times \frac{100}{E_{i}} \end{array}$$

The LIM score is the mean percentage of the maximal recommended values of three negative nutrients, i.e., sodium, added sugars, and saturated fatty acids. The LIM score for 100 g of food is calculated as following, where nutrient_il_ is the content (g, mg) of limited nutrient l in 100 g of food i, and MRV_l_ is daily maximal recommended value for nutrient l [54].

(3) $$\begin{array}{c} {LIM}_{i}=\frac{\sum_{l=1}^{l=3}{ratio}_{il}}{3} \end{array}$$

with

(4) $$\begin{array}{c} {ratio}_{il}=\left[\frac{{nutrient}_{il}}{M{RV}_{l}}\right]\times 100 \end{array}$$

By calculating the SAIN, LIM score, the food items are categorized in four classes: Recommended foods (class 1) if SAIN score is ≥5 and LIM score is <7.5, Neutral foods (class 2) if SAIN score is <5 and LIM score is <7.5, Eat less of these foods (class 3) if SAIN score is ≥5 and LIM score is ≥7.5, and Limit these foods (class 4) if SAIN score is <5 and LIM score is ≥7.5 [54].

The SAIN, LIM recommendations quantify the overall quality of individual’s dietary consumption. Thereby, the SAIN, LIM diet quality indicator combined with diet diversity indicators would ensure diversifying the diet with healthy foods, aiding to fulfill the nutritional requirements of every individual.

### 2.3. Design and Architecture of the ‘Diet DQ Tracker’

The ‘Diet DQ Tracker’ was designed as a self-administered smartphone app that could potentially replace traditional method of dietary data collection, such as in-person interviews via pen and paper questionnaire. The ‘Diet DQ Tracker’ aids in overcoming the limitations of traditional methods by switching to iOS or Android-based mobile devices. The app is compatible with iOS 9.3.5 and higher as well as Android 7.0 and higher.

**Figure 1:**
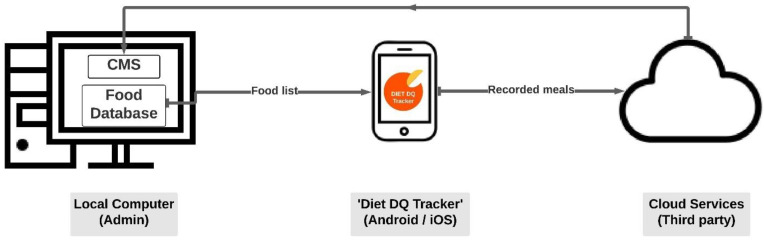
‘Diet DQ Tracker’ architecture.

The app’s primary objectives are: (1) To collect dietary data that can be used to assist intervention programs in resource-poor settings and (2) to facilitate self-monitoring of diet via real-time feedback based on the diet diversity indicators (MDD-W, IYCF-MDD, and HDDS) and the nutrient profiling system (SAIN, LIM). An extensive food and beverage database was developed, integrated, and stored in the third-party cloud services and linked with the app. In the database, the ingredients of food items are classified according to different food group categories of dietary indicators. The nutritional composition of food items contains the amount of energy, protein, fiber, calcium, iron, vitamin c, vitamin d, added sugar, saturated fatty acids, and sodium, aiding in calculating the SAIN, LIM score. The app provides real-time dietary feedback even in the absence of cellular data or wi-fi by permanently storing the food database imported from third-party cloud services in the app. After the user enters food items into the search-as-you-type text box, the database enables the app to perform calculations in the backend and generates the output score. If the app is connected to the internet, dietary data logged in the app are transferred to the AWS cloud; however, in the absence of internet, both input and output data are stored in the app, allowing the app to perform its core functions even in remote areas with limited or no internet access. The data from user’s ‘Diet DQ Tracker’ account are encrypted and transferred from the AWS cloud to the desktop-based local content management system (CMS) app, which is managed by an administrator. The dietary data present in research administrator’s CMS app can be exported as a comma-separated values file (CSV) for detailed analysis. The architecture of ‘Diet DQ Tracker’ is depicted in Figure 1.

**Figure 2:**
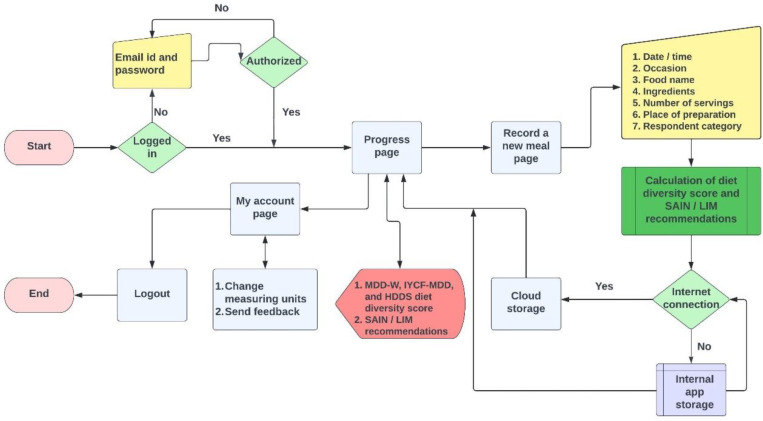
‘Diet DQ Tracker’ algorithm framework.

The structure of the ‘Diet DQ Tracker’ algorithm is illustrated in Figure 2. The algorithm was coded in the Python programming language, and it is available at the ‘Food Engineering Lab’ of McGill University, Macdonald Campus. If the user is already logged in upon app launch, the algorithm directs them to the progress page. The application would require a new user to manually enter their authorized email address and password. Users can view their MDD-W, IYCF-MDD, and HDDS diet diversity score (DDS) and SAIN, LIM recommendations from the progress page. To record a new meal, the user would have to enter the date/time, occasion, food name, ingredients, number of servings, place of preparation, and respondent category. After inputting all required meal characteristics, the algorithm will calculate the DDSs and SAIN, LIM recommendations (Formulas (1)–(4) in Section 2.2.1) using a predefined process based on the respective indicator guidelines. Following the data entry and calculation of the score, data are transferred to a third-party cloud storage in the presence of the internet; otherwise, they would be stored in the application’s internal storage. On the progress page, users can view the data input (meal characteristics) and data output (score achieved) in real time. From the ‘Progress’ page, users can access ‘My account’ to change the food quantity measuring units, provide feedback to the research team, and logout of the application.

The user-friendly design of the ‘Diet DQ Tracker’ incorporated with MDD-W, IYCF-MDD, HDDS, and SAIN, LIM aims to improve the diet diversity and overall diet quality in resource-poor settings. The app incorporated with a robust food database and CMS can collect, compute, and analyze real-time dietary intake by overcoming the limitations in traditional methodologies.

### 2.4. Food Database

A comprehensive database of foods and beverages from around the world linked to ‘Diet DQ Tracker’ is the core strength of the app. The automated process of coding and the score calculation empowered by the food database generates the output results simultaneously. This reduces the need of interviewer training for data collection and ensures the data collection accuracy. The USDA’s Food and Nutrient Database for Dietary Studies (FNDDS; 2017–2018), Laos’s ASEAN food composition database (electronic version; 2014-01-01), and Ethiopia’s food composition table for use in Ethiopia Part III (1968–1997) were used as the starting point of the ‘Diet DQ Tracker’ food database. The database accuracy has been enhanced by the inclusion of a wide range of verified macronutrients, micronutrients, and other food components from national food composition databases. The USDA, Laos, and Ethiopian databases contain 7083, 619, and 1075 foods, respectively. Currently, the app’s food database contains 8777 food products, both raw and cooked (steamed, boiled, roasted, fried, etc.), from a variety of categories such as cereals, starchy roots and tubers, legumes, nuts and seeds, vegetables, fruits, eggs, dairy, fat and oil, sugar, condiments and spices, snack foods (commercially processed and packed), fast foods (franchise foods), mixed food dishes, and alcoholic/non-alcoholic beverages. The food products and dishes were disintegrated into constituent ingredients and categorized into respective food groups as per MDD-W, IYCF-MDD, and HDDS scoring systems. Following this, these data were uploaded via CMS to the cloud. Nutritional values for energy, protein, fiber, calcium, iron, vitamin c, vitamin d, added sugars, saturated fatty acids, and sodium are provided per 100 g of food in the database. For Laotian and Ethiopian databases, the values of any missing nutrients were gathered from verified sources. To populate the database with local and indigenous food items from different socio-demographic backgrounds, users can add them via their ‘Diet DQ Tracker’ account. These foods can only be accessed by the individual in their local app storage and by research team in the CMS. After the approval of food groups and nutrient composition of food items, they are finally updated into the global database by the research team. The database is continuously maintained and updated by the research team.

The food database linked with the ‘Diet DQ Tracker’ aids in overcoming the limitations of traditional methodologies. It reduces the effort, time, and resources necessary for data collection by minimizing reliance on the interviewer’s skill and experience.

### 2.5. Content Management System

**Figure 3:**
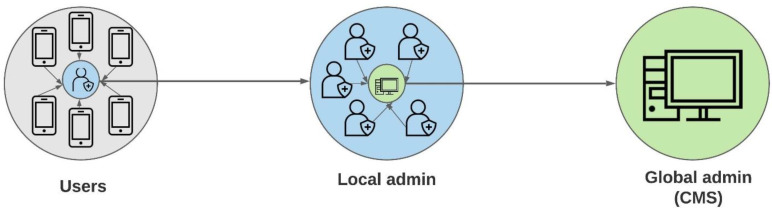
‘Diet DQ Tracker’ network.

The content management system (CMS) is the final stage to store and manage digital data from food database and user’s ‘Diet DQ Tracker’ account. It manages the increasing digital content collected from the national food composition tables and the end users. The CMS aids in the long-term cost-effective transition from a physical library to a hybrid of physical and digital libraries. An ideal CMS preserves, organizes, and disseminates both locally created and externally collected data, along with their associated metadata [55]. It is a desktop software that is used to manage the development and alteration of digital content from the smartphone app. Implementing the CMS with the ‘Diet DQ Tracker’ increases the information accuracy and flexibility and improves system management at a lower maintenance cost. The CMS empowers the global admin from research team with complete access to detailed information. Thus, the global admin is entitled with the ability to form local admins, representing the end-users of a specific region. Local admins are provided in-person and online training on CMS by global admin. Local admins are assigned auto-generated codes from the CMS as their unique identifier, specifying their region of administration. Global admins are in power of granting CMS access to local admins and end-users. The ‘CODES’ tab of global admin’s CMS version is used to manage local admins. Login accounts of end users are set up by global admin or local admin (if access granted by global admin) in the CMS by entering the end-user’s name, birth year, and email id, followed by assigning a password for the ‘Diet DQ Tracker’ app account. Following the account creation, these details are shared with end users, who can log into their accounts via the ‘Diet DQ Tracker’ app on their smartphones to record and save their dietary data in a central location accessible to global or local admins. End users can access their desktop-based CMS account from the same app account if a global or local admin grants permission. The login details of the end users can be accessed and modified by global or local admin from the ‘USERS’ tab of their CMS version. All CMS users can access the ’Diet DQ’ tab, which has three additional tabs, including ‘Meals added’, ‘Food list’, and ‘Ingredients’. Each row in the ‘Meals added’ tab is a unique entry which contains all the information on ‘user’, ‘local admin’, ‘mealtime’, ‘occasion’, ‘food name’, ‘serving’, ‘category’, and ‘prepared at’ recorded in the app, along with MDD-W, IYCF-MDD, and HDDS scores generated from this meal entry. All food items and beverages in the app database are present in the ‘Food List’ Tab. The ‘Food List’ tab provides information on the nutrient composition and food group categories according to different diet diversity indicators. All data stored in the CMS can be exported as an external file for further analysis. The ‘Diet DQ Tracker’ network diagram is demonstrated in Figure 3.

CMS enhances the data collection process by automating the data transfer while reducing the dependency on the skills of interviewers. Thus, CMS empowers the app to perform its prime functions with enhanced accuracy and correctness while overcoming the challenges encountered in traditional methodologies.

### 2.6. Pilot Study

The purpose of pilot validation study was to determine the acceptability, feasibility, and relative validity of the ‘Diet DQ Tracker’ by self-administered real-time food recording via app in comparison to the traditional methodology of interviewer-administered 24 h dietary recall. The aim was to compare the mean DDS at the group level with both methodologies and report on user preferences and experiences. It was hypothesized that the DDS would demonstrate moderate consistency between the two methodologies. The pilot study collected dietary data via both ‘Diet DQ Tracker’ and 24 h recall. The user experience with the ‘Diet DQ Tracker’ was collected by a participant feedback survey.

#### 2.6.1. Study Sample and Recruitment

The study sample constituted ten eligible participants for MDD-W and ten for HDDS dietary data collection. After recruitment, these twenty participants were questioned about their full name, email address, and birth year to generate their app account from the CMS application. Following the account generation, the app installation link and account login details were shared with the participants. One week prior to the data collection, participants received oral instructions on goals, features, and usage of the ‘Diet DQ Tracker’. Since one of the objectives was to ascertain the app’s usability and learnability, participants received instructions on the app’s basic functionalities rather than specific training on app usability.

#### 2.6.2. Dietary Assessment

Dietary assessment was performed to evaluate the performance of the ‘Diet DQ Tracker’ as a data collection tool. It was assessed by collecting data with the ‘Diet DQ Tracker’ and a 24 h recall for the same day. To collect data on an overlapping day, 24 h recall was administered the next day, capturing data for the same day the app was used.

‘Diet DQ Tracker’: Participants were instructed to record food items/beverages in ‘Diet DQ Tracker’ right after consumption for three fixed consecutive days (two weekdays and one weekend day). A daily reminder was sent for recording meals in the app. Weighing of food items was encouraged to record the number of servings; however, it was not a mandatory part of the procedure. Further, participants were directed to breakdown food recipes into their constituents and add them as single items under the ingredients. This was also followed by the interviewer during the 24 h recalls. Data collected over this three-day period was securely uploaded to cloud services by the app, which was accessible by CMS for detailed analysis.

24 h recall (reference measure): The interviewer-administered 24 h recall as a reference measure minimizes the correlation errors between the two methodologies. Participants were randomly contacted for unannounced dietary recall throughout the three-day period. Standard questionnaires with open-ended questions were developed for both MDD-W and HDDS.

For accurate dietary assessment, participants were strongly advised against relying on ‘Diet DQ Tracker’s recorded data for the previous day, to aid in remembering the food items consumed, during the 24 h recall.

#### 2.6.3. Participant Feedback on Dietary Assessment

The participant feedback on dietary assessment was collected to explore the participant experience with both methodologies. The participants completed a user feedback survey questionnaire on their experience with the app and a 24 h recall, including their preferred methodology, convenience, ease of use, time consumption, and learnability. Participants responded on statements using a 5-point Likert scale (strongly agree, agree, neutral, disagree, or strongly disagree). It was a vital process to learn about ‘Diet DQ Tracker’s acceptability, which would help improving the subsequent versions of the app.

## 3. Results

### 3.1. Features of ‘Diet DQ Tracker’

**Figure 4:**
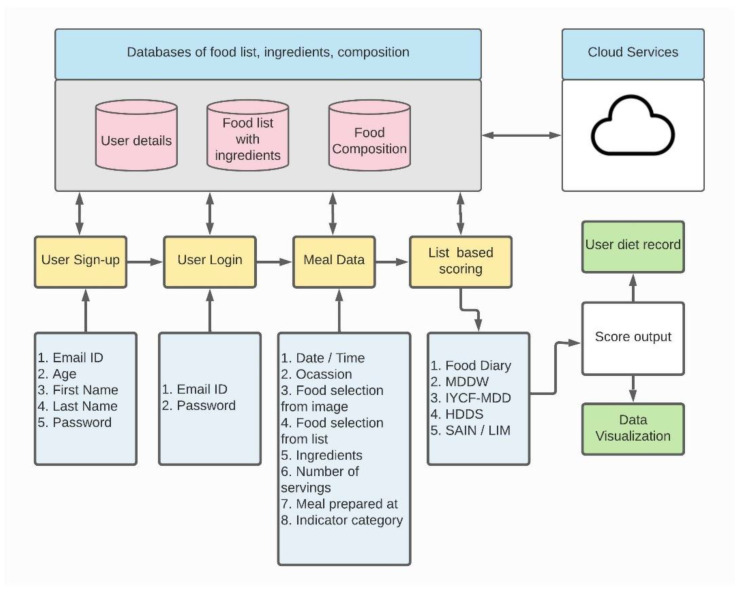
Functional chart of ‘Diet DQ Tracker’.

**Figure 5:**
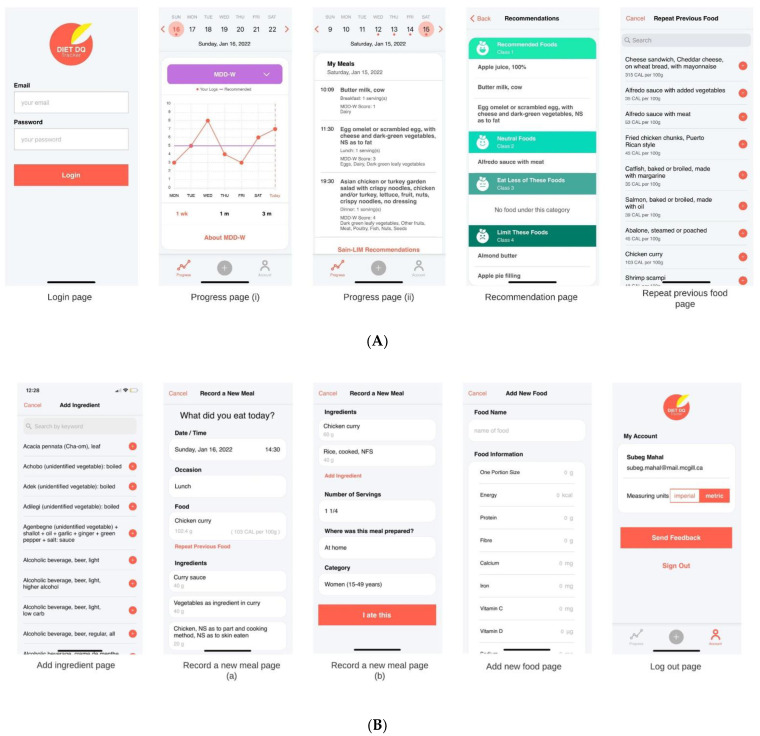
(**A**,**B**) Screenshots of ‘Diet DQ Tracker’.

‘Diet DQ Tracker’ was developed to help collect dietary information in an easy-to-use manner by overcoming the limitations imposed by traditional methodologies. The application has been developed over the course of 11 months with funding from the International Fund for Agricultural Development (IFAD). The functional flow chart for the ‘Diet DQ Tracker’ mechanism is visualized in Figure 4. The user interface comprises eight categories: ‘Login’, ‘My account’, ‘Progress’, ‘Recommendations’, ‘Record a new meal’, ‘Add a new food’, ‘Repeat previous food’, and ‘Add ingredient’. Figure 5A,B illustrate the screenshots of ‘Diet DQ Tracker’. Table 1 summarizes the components and functionalities of ‘Diet DQ Tracker’.

#### 3.1.1. Account Creation and Login

The account creation, followed by login, is the first step for data collection. To begin the process of creating an account, new users must provide their name, birth year, email, and password to their local admin. Once the account has been formed, users can access ‘Diet DQ Tracker’ via their smartphone by entering their designated email address and password on the login page of the app.

#### 3.1.2. ‘Progress’ Page

Progress page is the homepage for a logged in user. On the ‘Progress’ page, the default date at the top is set to current date. However, users can view their progress on any previous date by changing the pre-set default date. Beneath the date, ‘Progress’ page displays diet diversity scores per week, month, and three months in the form of a line chart, followed by basic indicator information after selecting one of the MDD-W, IYCF-MDD, or HDDS dietary diversity indicators. Below the line chart, a list of foods consumed on that date is displayed under ‘My meals’, along with other dietary data such as meal occasion, serving size, diet diversity score, and food groups. The main purpose of homepage is for users to simultaneously track their diet diversity scores and meal characteristics.

#### 3.1.3. ‘SAIN, LIM Recommendations’

The SAIN, LIM recommendations categorize user’s dietary intake into four classes. The recommendations on foods consumed in the last week can be accessed by clicking on SAIN, LIM Recommendations, which will take users to the ‘Recommendation’ page. On the ’Recommendation’ page, food items and beverages consumed are divided into four classes based on the SAIN, LIM score: ‘Recommended foods (Class 1)’, ‘Neutral foods (Class 2)’, ‘Eat less of these foods (Class 3)’, and ‘Limit these foods (Class 4)’. It prompts users to diversify their diets with healthy foods.

#### 3.1.4. ‘My Account’ Page

The ‘My account’ page help’s users manage their own personal account. At the bottom of the ‘Progress’ page, users can click on the ‘Account’ button for ‘My account’ page. On the ‘My account’ page, users can view their account details, send feedback to the global admin, and sign out of the application.

#### 3.1.5. ‘Record a New Meal’ Page

The ‘Record a new meal’ page probes users about various aspects of dietary intake. The users begin the dietary recording on this page by selecting the meal’s date/time and occasion. By default, the date and time fields for recording meals are set to the current date and time. Clicking on it, however, will allow the user to enter a meal for any previous date or time. After that, the user clicks on ‘Select occasion’ to record the meal occasion amongst: ‘Breakfast’, ‘Lunch’, ‘Dinner’, and ‘Snack’. Following that, the user clicks on the ‘Food item’ to record the food name and automatically moves to the ‘Add Food’ page. On the ‘Add Food’ page, the user enters the food or beverage consumed via the search-as-you-type text box. As soon as the user selects the food consumed, they need to enter the number of standard servings of that food item. After entering the number of servings, the user needs to specify the place of preparation of food, such as: ‘At home’, ‘At a restaurant’, and ‘Other’. This is followed by selecting the category of user that consumed the food, such as: Children (6–23 months), Women (15–49 years), and Household to record the meal for IYCF-MDD, MDD-W, and HDDS, respectively. In the case of IYCF-MDD, the ‘user’ refers to the mother or caretaker of the child. After entering all the necessary details, the user clicks on ‘I ate this’ button at bottom of the page to save the food consumed in the app database and update the dietary feedback on the progress page. This page accounts for the major proportion of time spent by users on the app. It has been designed for collecting all required meal characteristics in an efficient manner.

#### 3.1.6. ‘Add New Food’ Page

The ‘Add new food’ page facilitates users to add food items that are not present in the food database. The user can click on ‘New Food’ at bottom of the ‘Add Food’ page to access the ‘Add new food’ page. This page requests the food’s name and additional information such as portion size and associated energy, protein, fiber, calcium, iron, vitamin c, vitamin d, sodium, saturated fatty acid, and sugar content. To save this new food, the user clicks on ‘Add new food’ at the bottom, and this new food is saved in user’s local database in the app. Likewise, users can add new ingredients to the food consumed. The global admin checks the details of new food or ingredients, and finally updates the food database for all users. Additionally, ‘Diet DQ Tracker’ saves the previous food names consumed frequently by the user and provides the option to repeat it on the ‘Record a new meal’ page. This page helps in populating the food database with region specific indigenous and local foods.

### 3.2. Comparison of DDS’s from ‘Diet DQ Tracker’ and 24 h Recall

The statistical analysis figured out the limit of agreement and observed the difference in dietary scores generated by both methodologies. The DDS of individual respondents generated by the app was compared with DDS of the same individual, for the same day, generated manually by an interviewer administering 24 h recall. The DDS from these paired observations were compared by Wilcoxon sign-rank test. The *p*-values for MDD-W and HDDS DDS were 0.8501 and 0.7651, respectively, indicating no significant difference between the two methodologies.

**Figure 6:**
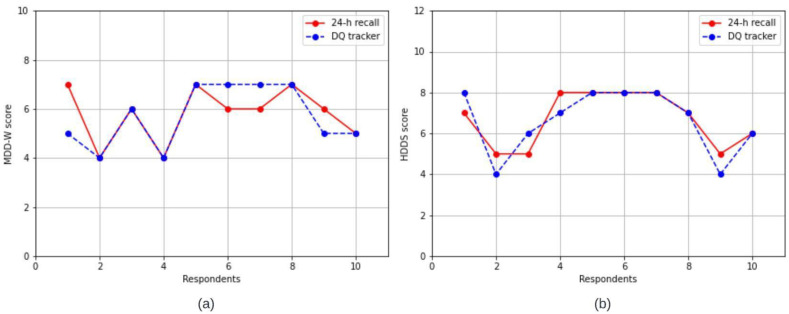
Line chart for (**a**) MDD-W and (**b**) HDDS representing DDS generated via 24 h recall and ‘Diet DQ Tracker’.

**Figure 7:**
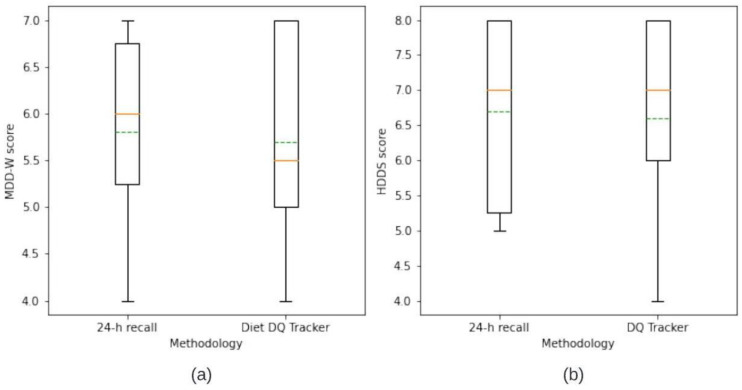
Box and whisker plot for (**a**) MDD-W and (**b**) HDDS representing DDS at group level for 24 h recall and ‘Diet DQ Tracker’.

**Figure 8:**
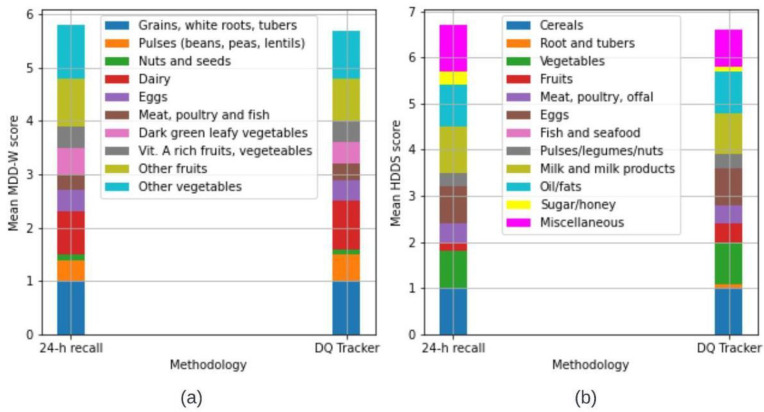
Stacked bar graphs for (**a**) MDD-W and (**b**) HDDS representing consumption of different food groups.

Respondent-wise, the DDS for MDD-W and HDDS by both ‘Diet DQ Tracker’ and 24 h recall is depicted in Figure 6a and Figure 6b, respectively. For MDD-W, six respondents had the same DDS via both methodologies, two respondents had a higher score via ‘Diet DQ Tracker’, and the remaining two respondents had a higher score via 24 h dietary recall. For HDDS, five respondents had the same DDS via both methodologies, two respondents had a higher score via ‘Diet DQ Tracker’, and three respondents had a higher score via 24 h dietary recall. The minimum, first quartile, mean, median, third quartile, and maximum of DDSs of MDD-W, and HDDS for ‘Diet DQ Tracker’ and 24 h recall were represented in box and whisker plots in Figure 7. Figure 8a,b illustrate the variation in the contribution of food groups to the mean MDD-W and HDDS scores, respectively, using both methodologies. The minimal difference in food group’s consumption demonstrated no significant variation between the data collected via both methodologies.

Overall, the results demonstrated insignificant difference in the DDS by ‘Diet DQ Tracker’ and the 24 h interviewer-administered dietary recall, indicating the potential to replace the traditional methodology.

### 3.3. Participant Feedback on ‘Diet DQ Tracker’ and 24 h Recall

The user experiences with the ‘Diet DQ Tracker’ and a 24 h telephone recall administered by an interviewer were gathered through the participant feedback survey. It has been summarized in Appendix A. In Table A1, majority of participants agreed or strongly agreed that the ‘Diet DQ Tracker’ was easy to understand (95%) and was easy to use (95%); foods and beverages were recorded on the app right after consumption (55%); and the app had proved to be highly satisfactory (90%). In addition, 95% of participants frequently accessed graphic visualizations of their MDD-W and HDDS scores, and 80% accessed SAIN, LIM food consumption recommendations on a regular basis. The graphic visualizations and SAIN, LIM recommendations by the app prompted consumption of diverse diets and healthy foods in 75% of MDD-W participants and 80% HDDS participants. In Table A2, ‘Diet DQ Tracker’ was preferred by all twenty participants for being convenient, easy to use, less time consuming, portable, and enjoyable to use.

Further, the average respondent time spent recording dietary data for single day in a 24 h period was 8.4 min with ‘Diet DQ Tracker’ and 10.25 min with 24 h recall. Apart from respondent’s time, interviewers spent approximately 22 to 25 min per respondent via 24 h dietary recall. This duration totals time spent on manually categorizing ingredients into respective food groups and feeding data into a computer for analysis. Thus, total respondent and interviewer time spent via 24 h recall was 32.25 to 35.35 min, which is roughly equivalent to half a man-hour. On the contrary, the automated process of data collection and cloud storage by the app significantly reduced interviewer burden. It was deduced that, on average, dietary data from seven respondents could be collected via ‘Diet DQ Tracker’ in one man hour, representing nearly a 400% increase in the number of respondents.

Therefore, the ‘Diet DQ Tracker’ was highly acceptable to the participants over the traditional methodology. In addition, the app exhibited potential to collect data from larger study samples in less time than traditional methodologies.

## 4. Discussion

### 4.1. Principal Considerations

The purpose of this study was to introduce the design and development of ‘Diet DQ Tracker’. Automating the collection of food consumption data and the coding of foods and portion sizes are some of the new technologies that are being used in the field [9,22]. The app with its robust food database from different nationalities and cultures tends to augment dietary assessment in nutrition sensitive agriculture by potentially replacing conventional methodology of interviewer-administered questionnaire. In addition to reducing the workload of the researcher, ‘Diet DQ Tracker’ probes different aspects of meal intake from users in their own time. The app can be downloaded and installed by participants on their personal smartphones, thus saving research resources while increasing the geographic reach of data collection. ‘Diet DQ Tracker’s features include real-time meal recording from a list of thousands of pre-defined foods and beverages, personalized dietary diversity feedback, and recommendations based on SAIN, LIM nutrient profiling system. All meal entries of a particular date are analyzed and visualized collectively, followed by permanent cloud storage that can be retrieved via CMS. Throughout the development process, a balance was struck between the researcher’s desire to collect detailed and accurate dietary assessment data and the user’s desire to spend minimal time with the tool.

### 4.2. Strengths and Limitations

‘Diet DQ Tracker’ has multiple strengths which were identified during the development and validation study. These strengths provide it with an edge over the traditional methodologies. The primary strength is that it is the first smartphone application designed to collect data for globally recognized diet diversity indicators, such as MDD-W, IYCF-MDD, and HDDS used in resource-poor settings. Unlike traditional methods, the app is designed to collect dietary information immediately after consumption and thus does not rely on human memory. Thus, by shifting from recall approach to record-like approach for data collection, ‘Diet DQ Tracker’ overcomes recall bias, as experienced in traditional methodologies. Further, the personal respondent account on ‘Diet DQ Tracker’ ensures privacy and eliminates the need for respondent-interviewer interactions, thus overcoming the social-desirability bias observed with traditional methodologies. Further, respondents being preoccupied with other tasks may systematically disregard traditional time-consuming surveys. The fact that 100% of respondents preferred to provide their dietary data via ‘Diet DQ Tracker’ demonstrated the app’s acceptability over traditional interviewer-administered 24 h dietary recalls. ‘Diet DQ Tracker’ helped in speeding up the data collection and analysis process. The results from pilot study determined that, on average, seven respondent’s dietary data could be collected and analyzed via app in one man hour. This represents a 400% increase in the number of respondents in comparison to a 24 h dietary recall. Additionally, since no significant supplementary cost is required to expand the number of respondents or data entries, ‘Diet DQ Tracker’ can collect dietary data from large study samples, for longer periods of time compared to traditional methodologies.

The application incorporates a novel and extensive food composition database that generates the score for indicators and suggests the food consumption by the SAIN, LIM nutrient profiling system in real time. The ‘Diet DQ Tracker’ food database with nutritional content of food items significantly reduced the interviewer burden. Consequently, it demonstrated minimal dependency on the interviewer’s skill and ability, which are necessary for a 24 h recall. The desktop-based CMS application enables the researcher to add, delete, and edit food items and their associated nutrient content in response to user demand and recent food composition table updates. The application serves three distinct target audiences (women of reproductive age, infants and children under the age of two, and households, including all members) and is user-friendly across a broader range of the population. The application gives real-time dietary feedback by computing the scores in backend (without additional coding). The data collected by the app is automatically stored in the cloud and represented in the CMS application for further analysis.

All these strengths combine to cover the gaps in traditional methodologies. Thus, ‘Diet DQ Tracker’ possesses the ability to replace conventional dietary data collection methodologies for diet diversity indicators in resource-poor settings.

On the other hand, a few constraints were encountered while designing, developing, and validating the first version of ‘Diet DQ Tracker’ against the interviewer-administered 24 h recall. Therefore, there is still some room for improvement. The first limitation was the user burden associated with recording dietary data from a vast list of food items and beverages. While the user is presented with a search-as-you-type text box, navigating through the vast food and beverage list can be challenging. However, the presence of the ‘Repeat previous food’ function alleviates some of this burden by prioritizing a user’s list of frequently consumed food items. Commercial apps may have a concise list of food items, but this is likely to exclude foods that are not frequently consumed by the population. Additionally, they are likely to result in less precise food records and nutrient intake calculations [34]. Further, the user interface in current version of app supports English language, which might hinder the usability in resource-poor settings with lower literacy levels and different cultures. Considering the app’s complexity in terms of indicators, food database, and score calculation, challenges encountered while collaborating with an external software company included online video meetings (due to COVID-19 pandemic), communication, and effective project management. A few of these issues have been identified frequently when nutrition researchers team up with external software development companies. Furthermore, keeping an up-to-date database of food compositions will be a continuous challenge given the frequency with which food and beverage manufacturers reinvent products or introduce new ones in the market.

Another limitation in current pilot study, typically in validation or comparative studies [33,56], is that recording food items in ‘Diet DQ Tracker’ may have influenced the interviewer-administered 24 h recall (next day) by reducing memory bias. Additionally, access to ‘My meals’ on the ‘Progress page’ might have helped respondents in recalling food items during 24 h recall. The majority of participants strongly agreed that ‘My meals’ were checked during the dietary recall. It is one prime reason for the reduced duration of 24 h recalls (10.25 min) compared to other validation studies. In validation study of e-DIA, it took 30 min to record food items via interviewer-administered 24 h telephone recall [34]. Further, most participants were under the age of 35 and expressed high levels of comfort using the app on their smartphones. The quality of dietary data collected via smartphone or computer-based dietary assessment tools is directly proportional to the educational level [57] and inversely proportional to the age group [58]. Therefore, caution is necessary while extrapolating current findings to the general population.

These limitations will be addressed in future studies to further enhance the app’s dietary assessment and user acceptability. Large scale validation studies will be conducted to further explore these limitations. These limitations will be taken into account during the design and development of ‘Diet DQ Tracker’s subsequent versions.

### 4.3. Related Work

Prior studies have found a high level of agreement between traditional and modern approaches, with the latter being preferred by most participants [59]. A feasibility study of a commercial smartphone app compared a 4-day food diary completed by a modified version of ‘Easy Diet Diary’ with two 24 h dietary recalls performed during the same week [23]. The app was preferred by 50 adults (82 per cent of whom were women) for completing 24 h dietary recalls. The average energy consumption discrepancy between the traditional method and the smartphone app was 268 kJ/d, and most of the limits of agreement were within the acceptable range. In another validation study, the ‘Eat and Track’ (‘EaT’) app was used to track participant’s food and beverage intake for 3 days, and the results were compared with three dietitian-administered 24 h recall interviews conducted on the same days as the reference method [33]. Although the median energy intake was significantly different between the two methods, the ‘EaT’ app had acceptable agreement for the majority of nutrient densities at the group level. Evaluation study of ‘e-CA’, an electronic mobile-based food record had good agreement with 24 h dietary recalls but confirmed the complexity of determining the portion sizes [26]. Another validation study comparing mobile phone app ‘e-DIA’ with 24 h dietary recall found no significant differences in mean energy or nutrient intakes between the two methods [34]. In another validation study of ‘My meal mate’, a weight loss app, revealed wide limits of agreement with 24 h recall at the individual level but demonstrated great potential as a group-level dietary assessment tool [27]. Although, ‘Diet DQ Tracker’ is the first smartphone app designed to collect dietary data for diet diversity indicators, future studies will aim at evaluating the performance of ‘Diet DQ Tracker’ with the other smartphone applications.

### 4.4. Future Work

The future work will be based on the limitations experienced in the current pilot study. The selection of biological methods such as doubly labeled water (DLW) as a reference method for the validation of diet assessment apps may be challenging due to the complexity, cost implications, and requirements of highly controlled settings [60]. Further, the traditional methodologies have inherent limitations that effect the validation of diet assessment, when compared with mobile-based apps [60]. However, the performance of ‘Diet DQ Tracker’ in comparison to traditional, biological, and modern technology-based methods needs to be investigated in large-scale studies involving respondents of various population groups. A new feature will be added that incorporates food recognition by taking a picture of the food item via smartphone camera. The app will be intelligent enough to recognize the food and provide real-time dietary feedback. The app’s database stored in cloud will be updated with hundreds of images of individual food items aiding the algorithm in food identification. Facilitating respondents with food portion-size booklets or standard weighing scales could potentially improve accuracy of diet quality scores. The future versions of the app will also be developed in different languages, allowing it to reach respondents from different cultural backgrounds, especially in resource-poor settings. Additionally, food composition tables containing a variety of local and indigenous foods representing multiple nationalities will be added on a regular basis to strengthen the database. The system will be maintained periodically to ensure that the food database remains up to date. Thus, there are still potential opportunities for improvement that will be targeted in the subsequent validation studies of the app.

## 5. Conclusions

The main objective of the current study was to demonstrate the design and development of smartphone app ‘Diet DQ Tracker’. The app has been developed to overcome the challenges encountered in collecting dietary data for diet diversity indicators via traditional methodologies, such as 24 h interviewer-administered dietary recalls. The pilot study demonstrated nearly identical mean DDS’s at the group level via both methodologies. The app, being more convenient, easy to use, less time-consuming, portable, and enjoyable to use, was preferred by all participants over interviewer-administered 24 h recall. The app reduced the respondent–researcher burden, facilitated data collection by cost- and time-efficient methodology, and demonstrated high respondent acceptability. However, the sample size of twenty participants was a significant limitation in the current pilot study. Therefore, the performance of the ‘Diet DQ Tracker’ will be evaluated in large scale studies involving respondents from different age groups with diverse socio-demographic and educational backgrounds. Further, subsequent versions of app will be continually populated with different local and indigenous foods from resource-poor settings, accessible in local languages.

## Figures and Tables

**Table 1:** Components and functionalities of ‘Diet DQ Tracker’.

	Components of ‘Diet DQ Tracker’	Description of Functionalities
1.	‘Progress’ page	To view the progress of the user by selecting a date To view the graphical representation of diet diversity scores by selecting ‘MDD-W’, ‘IYCF-MDD’, or ‘HDDS’ To view the visual representation over 1 week, 1 month, or 3 months by selecting ‘1 wk’, ‘1 m’, or ‘3 m’ To view the background information of indicators by clicking on ‘About MDD-W’, ‘About IYCF-MDD’, or ‘About HDDS’ To view the meals consumed on the selected date by scrolling down to ‘My meals’ To view the SAIN, LIM recommendations of foods consumed over the past week by selecting ‘SAIN-LIM Recommendations’
2.	‘Record a new meal’ page	To specify the date and time of meal consumption by clicking on pre-set date/time To specify the occasion of meal consumption (breakfast, lunch, dinner, and snack) by clicking on ‘Select occasion’ To specify the food name by using the search-as-you-type box after clicking on ‘Food item’ To specify the food name from food items frequently consumed by the user by clicking on ‘Repeat previous food’ To add a new food to the database by clicking on ‘Add new food’ at the bottom of ‘Add Food’ page To add ingredients to the selected food item by clicking on ‘Add ingredient’ To specify the number of servings of the food item by clicking on ‘Select number of servings’ To specify the place of meal preparation (at home, at a restaurant, other) by clicking on ‘Select meal prepared at’ To specify the category of user (children, women, or household) who consumed the meal by clicking on ‘Select your category’ To upload the data by clicking on ‘I ate this’
3.	‘My account’ page	To change the units of food portion size on the ‘Record a new meal’ page from imperial to metric by clicking on ‘Measuring units’ To send the feedback on any issue/problem/or suggestion to the research team by clicking on ‘Send feedback’ To sign out of the application by clicking on ‘Sign out’

## Data Availability

Not applicable.

## Appendix A

**Table A1:** Participant feedback on usability of ‘Diet DQ Tracker’ and 24 h Recall.

User Feedback on Dietary Assessments				
Number of Respondents		20 (10 Female; 10 Male)		
1.	General statements To begin with the survey, I’d like to read a few statements regarding the dietary survey in which you agreed to participate. I will read the statements for you; please choose one of the options below. Please select, whether you strongly agree, agree, are neutral, disagree, or strongly disagree with the following statements.	Agree/Strongly agree	Neutral	Disagree/Strongly disagree
1.1	The Diet DQ app is easy to understand.	19	1	
1.2	The Diet DQ app is easy to use.	19	1	
1.3	The foods I usually consume were easy to find on the app.	5	13	2
1.4	Foods and beverages were recorded on the app right after consumption.	11	9	
1.5	Recording food items right after consumption affected my daily routine.	1	7	12
1.6	During the interview administered recall session, remembering the food items consumed the previous day was difficult.	5	6	9
1.7	Meals logged the day before were checked prior to or during the interviewer-administered recall session.	13	4	3
1.8	Recording food items in the ‘Search-as-you-type’ textbox was convenient.	10	6	4
1.9	Estimating the number of servings of a standard portion size was easy.	6	5	9
1.10	Custom recipes were frequently recorded on the Diet DQ app.	8	7	5
1.11	Recording custom recipes on the app was difficult.	4	12	4
1.12	The ‘repeat previous food’ feature was often used.	2	7	11
1.13	Graphic visualizations of the diet diversity scores were frequently accessed.	19	1	
1.14	The graphic visualizations prompted the consumption of diverse food groups for the purpose of achieving a higher score.	15	5	
1.15	SAIN, LIM recommendations for foods consumed were frequently checked.	15	4	1
1.16	SAIN, LIM recommendations prompted the consumption of healthy foods.	16	4	
1.17	Overall, the Diet DQ app proved to be highly satisfactory.	18	2	

**Table A2:** Participant feedback on preferred methodology.

1.	The Preferred Methodology		
1.1	Which is your preferred methodology for dietary assessment?	App	Interview-Administered Recall
		20	
	Provide Yes or No answers below. Was your preferred dietary assessment methodology:	Yes/No	
1.2	More convenient?	Yes (20)	
1.3	Easy to use?	Yes (20)	
1.4	Less time consuming?	Yes (20)	
1.5	Easy to remember?	-	
1.6	Portable?	Yes (20)	
1.7	Enjoyable to use?	Yes (20)	
2.	Duration questions	Time (min)	
2.1	Approximately how long did it take to record an entire day’s food intake on the Diet DQ app?	8.4	
2.2	Approximately how long did it take to recall and inform the interviewer about the previous day’s food consumption?	10.25	
